# The m^6^A RNA methyltransferase METTL3/METTL14 promotes leukemogenesis through the mdm2/p53 pathway in acute myeloid leukemia

**DOI:** 10.7150/jca.60381

**Published:** 2022-01-04

**Authors:** Lina Sang, Xia Wu, Tianyou Yan, Duolan Naren, Xiaoyan Liu, Xue Zheng, Nanchen Zhang, Huifang Wang, Yarong Li, Yuping Gong

**Affiliations:** Department of Hematology, West China Hospital, Sichuan University, China, 610041.

**Keywords:** m6A RNA methylation modification, METTL3, METTL14, hematopoietic stem cell, acute myeloblastic leukemia

## Abstract

N6-methyladenosine (m^6^A) is the most abundant internal modification in mammalian mRNA and recent studies have highlighted the importance of m^6^A levels in tumor development. In this study, we investigated the expression of methyltransferase-like 3 (METTL3) and 14 (METTL14), components of the RNA m^6^A methyltransferase complex, in samples from 89 patients with acute myeloid leukemia (AML), and followed the survival of 75 of these patients. Our results show that METTL3 and METTL14 are highly expressed in most of the patients with AML (except those with APL), and high levels of METTL3 and/or METTL14 correlated to shorter survival in the patients. In leukemia cell lines K562 and kasumi-1, both METTL3 and METTL14 promote cell proliferation and cell cycle, and the knockdown of METTL3 and METTL14 inhibits proliferation, and induces apoptosis and differentiation. Notably, the knockdown of METTL3 and METTL14 in K562 cell line leads to several changes in the expression of p53 signal pathway, including the upregulation of p53, cyclin dependent kinase inhibitor 1A (*CDKN1A*/*p21*), and downregulation of mdm2. Importantly, the m^6^A level of mdm2 mRNA was significant lower after knock-down of METTL3 and METTL14 examined by m^6^A-RIP and mdm2 qPCR assay, and the half-life of mdm2 under actinomycin-D treatment became shorter. Taken together, our study demonstrates that the lower m^6^A levels of mdm2 mRNA mediated by the knockdown of METTL3 and METTL14 could lead to the low stability of mdm2 mRNA transcripts and low expression of MDM2, in the end, activate p53 signal pathway. Both METTL3 and METTL14 play an oncogenic role in AML by targeting mdm2/p53 signal pathway.

## Introduction

Epigenetics refers to reversible and heritable modifications without changes in the nuclear DNA sequence. DNA methylation and histone modification are epigenetic modifications that play a very important role in cell biology and disease. Similarly, RNA methylation is also involved in the regulation of many biological processes. N6-methyladenosine (m^6^A) is the most abundant internal modification in mammalian mRNA and was first discovered in the 1970s 1-2. However, the importance of this epigenetic modification has been uncovered only recently. In mammals, the m^6^A modification is catalyzed by a multi-component methyltransferase complex, which includes methyltransferase-like 3 (METTL3) and 14 (METTL14) and their cofactor Wilms' tumor 1-associated protein (WTAP); on the other hand, m^6^A can be demethylated by two demethylases: fat mass and obesity-associated protein (FTO) and AlkB homologue 5 (ALKBH5) 3-7. Members of the YT521-B homology domain family (YTHDF), such as YTHDF1 and YTHDF2, are m^6^A binding proteins that mediate mRNA translation, stabilization, and alternative splicing 8-12. The m^6^A RNA modification is dynamic and reversible, and can affect a variety of biological processes, such as the maintenance and promotion of the differentiation of embryonic stem cells 13, regulation of the circadian rhythm 14, heat shock response after DNA damage 15, adipogenesis 16-17, spermatogenesis 18-19 and hematopoietic development 20-25.

In recent years, the relationship between METTL3-METTL14 and disease, especially tumors, has attracted much attention. In glioblastoma, METTL3 and METTL14 act as tumor suppressors, while FTO and ALKBH5 are oncogenes 26-27; on the contrary, it has been found that METTL3 promotes growth, invasion and translation in lung cancer cells, indicating that the roles of the m^6^A modification in tumors are complex 28. A clinical study has shown that WTAP is an oncogene in acute myelogenous leukemia (AML) 29; however, the function of WTAP as m^6^A methyltransferase cofactor in AML is unknown. FTO exerts its oncogenic role as an m^6^A demethylase by targeting critical transcripts such as ankyrin repeat and SOCS box protein 2 (*ASB2*) and retinoic acid receptor alpha (*RARA*) 30. Recently, it has been reported that METTL3 maintains the undifferentiated leukemic phenotype by targeting *c-MYC*, B-cell lymphoma 2 (*BCL2*) and phosphatase and tensin homolog (*PTEN*) mRNA 20. METTL14 is required for the development and self-renewal of leukemia stem/initiating cells by regulating the expression of genes such as *MYB* and *MYC* 21. Additionally, METTL3 is a regulator of a chromatin-based pathway and is necessary for the maintenance of the leukemic state by controlling m^6^A-dependent translation 24. These studies have shown that METTL3 and/or METTL14 play an oncogenic role in AML. However, the mechanism through which they exert their function in AML is unknown. Therefore, we decided to explore the functional role of the m^6^A RNA methyltransferases METTL3 and METTL14 in AML.

We collected bone marrow or peripheral blood samples from 89 patients with AML to detect the expression of METTL3 and METTL14 by quantitative PCR (qPCR) and Western Blot, and followed up the survival of 75 patients. We found that the expression of METTL3 and METTL14 was high in most of the patients with AML, and high expression of METTL3 and METTL14 in AML samples correlates with poor prognosis. Upon the knockdown of METTL3 and METTL14 in K562 and kasumi-1 cell lines, we also found that METTL3 and/or METTL14 promote leukemogenesis. Moreover, RNA-sequencing (RNA-seq) and m^6^A-RIP data revealed that METTL3 and METTL14 plays a crucial role in the development of leukemia by the mdm2/p53 signaling pathway.

## Materials and Methods

### Patient samples

The leukemic bone marrow or peripheral blood samples (leukemia blast cells >30%) from patients with AML were collected at the time of diagnosis or relapse in the West China Hospital, Sichuan University (China) from 2015 to 2017, and blood samples from healthy subjects were used as control. Written informed consent was obtained from the patients, and the study was approved by the local ethics committee. Mononuclear cells were isolated by Ficoll (TBD Science, Tianjin, China) density gradient centrifugation.

### Cell culture

Human chronic myelogenous leukemia (blast crisis) K562, acute myeloid leukemia Kasumi-1 and HL60, acute promyelocytic leukemia NB4, acute myelogenous monocytic leukemia MV4-11, acute monocytic leukemia THP-1, acute T lymphocyte leukemia CEM and MOLT4, and cervical tumor HEL cells were cultured in RPMI1640 medium (Hyclone, USA) supplemented with 10% FBS and 1% penicillin/streptomycin. Human bronchial epithelial HBE and embryonic kidney 293T cells were cultured in DMEM (Hyclone) supplemented with 10% FBS and 1% penicillin/streptomycin. HEL, CEM and MOLT4 cells were obtained from the Cell Bank of the Shanghai Academy of Science (Shanghai, China), the other cell lines were obtained from the hematology lab of the West China Hospital.

### Cell transfection

K562 cells were infected with lentiviral particles over-expressing short hairpin RNA (shRNA) targeting *METTL3* and *METTL14*. The sequence of the shRNA targeting *METTL3* was 5'-CCG GGC CAA GGA ACA ATCC ATT GTT CTC GAG AAC AAT GGA TTG TTC CTT GGC TTT TTG-3', the sequence of the shRNA targeting *METTL14* was 5'-CCG GCC ATG TAC TTA CAA GCC GAT ACT CGA GTA TCG GCT TGT AAG TAC AGG TTT TT-3'. The percentage of green fluorescent protein, also expressed by the lentiviral particles and representing transfection efficiency, was measured by flow cytometry 48h after infection. The knockdown efficiency of *METTL3* and *METTL14* was measured by qPCR and Western Blot two-three weeks after transfection. For the control cells, K562 and Kasumi-1-CTR cells were infected with a non-targeting shRNA (control lentivirus), respectively.

### qPCR

Total RNA was isolated with TRIzol (Invitrogen, Carlsbad, CA, USA) according to the manufacturer's protocol. Five hundred nanograms of RNA were reverse transcribed with PrimeScript™ RT Master Mix (Takara). qPCR was performed using Hieff™ qPCR SYBR^®^ Green Master Mix (yeasen, Shanghai, China) on a LightCycler^®^ 480 system (Roche). The primers used were as follows: glyceraldehyde 3-phosphate dehydrogenase (*GAPDH*) forward: 5'-TCAAGGCTGAGAACGGGAAG-3', reverse: 5'-TGG ACT CCA CGA CGT ACT CA-3'; *METTL3* forward: 5'-GAA GCA GCT GGA CTC TCT GC-3', reverse: 5'-ACG GAA GGT TGG AGA CAA TG-3'; *METTL14* forward: 5'-AAA GAG AGG CAG CGA CAA GA-3', reverse: 5'-TGA GGG TTT TCC GTA GTT GG-3'. *p21* forward:5'-TGG AGA CTC TCA GGG TCG AAA-3', reverse: 5'-GGC GTT TGG AGT GGT AGA AA C-3'. *ATM* forward:5'-CCG TGA TGA CCT GAG ACA AG-3', reverse: 5'-AAC ACC ACT TCG CTG AGA GAG-3'.* mdm2* forward: 5'-GGC AGG GGA GAG TGA TAC AGA-3', reverse: 5'- GAA GCC AAT TCT CAC GAA GGG-3'.

### Western Blot

Cells were lysed with radio immuno-precipitation assay buffer (Beyotime) containing a protease inhibitor cocktail (cOmplete™, Mini, EDTA-free, EDTA-free Protease Inhibitor Cocktail; Roche). The cell lysate was incubated on ice for 15min and centrifuged at 4 ºC for 20 min at 14000*g*. The protein concentration of the supernatant was measured using an enhanced BCA protein assay kit Enhanced BCA Protein Assay Kit Enhanced BCA Protein Assay Kit Enhanced BCA Protein Assay Kit (Beyotime). The denatured proteins were resolved by 10% sodium dodecyl sulfate-polyacrylamide gel electrophoresis under reducing conditions and transferred to polyvinylidenefluoride membranes (GE Healthcare). The membranes were incubated with primary antibodies at 4 °C overnight. After washing in TBST, the membranes were incubated with horseradish peroxidase-conjugated secondary antibodies and the immunosignals were visualized using the Super ECL Detection Reagent (yeasen, Shanghai). Primary antibodies were: rabbit anti-METTL3 (1:2000, Abcam), rabbit anti-METTL14 (1:2000, Sigma), mouse anti-GAPDH monoclonal antibody (1:5000, ZSGB-BIO).

### Proliferation assays

Cell viability assessed using a 3-(4,5-dimethylthiazol-2-yl)-2, 5-diph-enyltetrazolium bromide (MTT) assay kit (AMRESCO). Cells (5×10^3^ cells/well) were seeded into 96-well plates and cultured for 24, 48, 72and 96h. MTT (20μl from a 5mg/ml stock) was added to each well and the plates were incubated at 37ºC for 4h. At the end of incubation, a sodium dodecyl sulfate/isobutanol/HCl solution (100μl/well) was added. The absorbance was measured using a Multiskan™ FC (Thermo Scientific) at 570nm.

### Analysis of m^6^Alevels using dot-blot assays

mRNA was purified from total RNA twice using a GenElute mRNA miniprep kit (Sigma-Aldrich) according to the manufacturer's instructions. mRNA concentration was measured using Qubit (Invitrogen). Isolated mRNA was denatured by heating at 95 ºC for 3 min, and chilled on ice. Two-fold serial dilutions were spotted on nylon membranes (Roche). After UV cross-linking in a Stratagene Stratalinker 2400 UV cross-linker, the membrane was washed with PBST, blocked with 5% non-fat milk in PBST, and incubated with an anti-m^6^A antibody (1:1000, Abcam) at 4°C overnight. After incubation with horseradish peroxidase-conjugated anti-rabbit IgG secondary antibody (Jackson ImmunoResearch), the immunosignals was visualized using the ECL method. To ensure equal amounts of mRNA were spotted on the membrane, the blot was also stained with 0.02% methylene blue in 0.3 M sodium acetate (pH 5.2).

### Liquid chromatography with tandem mass spectrometry (LC-MS/MS)

mRNA (500 ng) was digested with nuclease P1(1U, Sigma) in 20μl of buffer containing 25mMNaCl and 2.5μM ZnCl_2_ at 37℃ for 4h; NH_4_HCO_3_(1M, 3μl) and alkaline phosphatase (0.25U) were then added. After incubation at 37℃ for 4h, the solution was used for LC-MS/MS 6. A triple quadruple mass spectrometer (AB sciextriplequan TM 5500, USA) equipped with a Shimadzu ultra-fast liquid chromatographic system was used to quantify m^6^A and A. The separation was performed on a Waters BEH C18 column (2.1 mm×100 mm, 1.7 mm). The samples were analyzed via multiple reaction monitoring with the transitions of m/z 282.0 to 150.0 for m^6^A, and m/z 136.1 to 119.1 for A. Quantification was performed by comparison with a standard curve, and the calculated concentrations were used to calculate the ratio of m6A to A.

### Colony formation assays

The anchorage-independent growth of the cells was estimated by colony formation assays on soft agar, as described 31. A single-cell suspension (1×10^3^ cells/well) was plated in 24-well plates in 500μl of RPMI1640 medium containing 10% FBS and 0.35% agar on a layer of 500μl of the same medium containing 0.5% agar. Two-three weeks after plating, the colonies were stained with 0.04% crystal violet-10% ethanol in ddH_2_O and photographs of the stained colonies were taken by a camera.

### Flow cytometry

Apoptosis rates were determined by Annexin V-647 and propidium iodide staining and cell cycle was detected by RNase A and propidium iodide staining according to the instructions of a Cell Cycle Analysis kit (yeasen). The percentage of CD14 and CD11b positive cells was examined by flow cytometry after the treatment with all-trans retinoic acid (ATRA; Sigma) or phorbol 12-myristate 13-acetate (PMA; yeasen). Flow cytometry was performed on a BD cytometer, and the data were analyzed with the FlowJo VX software.

### RNA-seq

Total RNA (3μg) was isolated from *METTL3* knockdown (shMETTL3), *METTL14* knockdown (shMETTL14) or control (K562-CTR) cells with two repetitions when the knockdown efficiency was measured by qPCR and Western Blot two weeks after transfection, mRNA was purified from total RNA using poly-T oligo-attached magnetic beads. Sequencing libraries were generated using NEBNext^®^Ultra™ RNA Library Prep Kit for Illumina^®^ (NEB, USA) following manufacturer's recommendations, and index codes were added to attribute sequences to each sample. The clustering of the index-coded samples was performed on acBot Cluster Generation System using TruSeq PE Cluster Kit v3-cBot-HS (Illumina) according to the manufacturer's instructions. After cluster generation, the library was sequenced on an Illumina Hiseq platform and 125/150 bp paired-end reads were generated. The HTSeq v0.6.1 software (Illumina) was used to count the reads for each gene. The fragments per kilobase of gene model per million mapped reads (FPKM) of each gene were calculated based on the length of the gene and reads count mapped. Differential expression analysis was performed using the DESeq R package (1.18.0).

### M6A-RIP/MeRIP

Total RNAs were extracted with TRI Reagent® (Molecular Research Center, Inc., USA). For m6A RIP, RNAs from control and WTAP knocked down cells were fragmented into around 300-nt fragments by incubation at 94 °C for 30s in fragmentation buffer (10 mM ZnCl2, 10 mM Tris-HCl, pH 7.0). The reaction was then stopped with 0.05 M EDTA (Ambion, AM8740), followed by standard ethanol precipitation and collection. Anti-m6A polyclonal antibody was purchased from Synaptic Systems. Magna RIPTM RNA-Binding Protein Immunoprecipitation Kit (Millipore, Germany) was used according to manufacturer's protocol. The mRNAs pulled down by anti-m^6^A antibody were then reversely transcribed and amplified following the protocol above.

### mRNA stability assays

K562 cells were incubated with 5μg/ml actinomycin D for 2, 4 or 6, and qPCR assays were conducted to quantify the relative levels of target mRNA. The degradation rate of target mRNA was estimated according to a previously published study 30.

### Statistical analysis

Individual experiments were conducted in triplicate. For statistical comparison, the Student's t-test was employed. Kaplan-Meier analysis and log-rank test were used to evaluate the differences in patient survival. Statistical analyses were performed using the IBM SPSS Statistics 24 software. Data were considered statistically significant as follows: *P<0.05, **P<0.01 and ***P<0.001.

## Results

### High expression of METTL3 and METTL14 correlates to shorter survival in patients with AML

**Figure 1:**
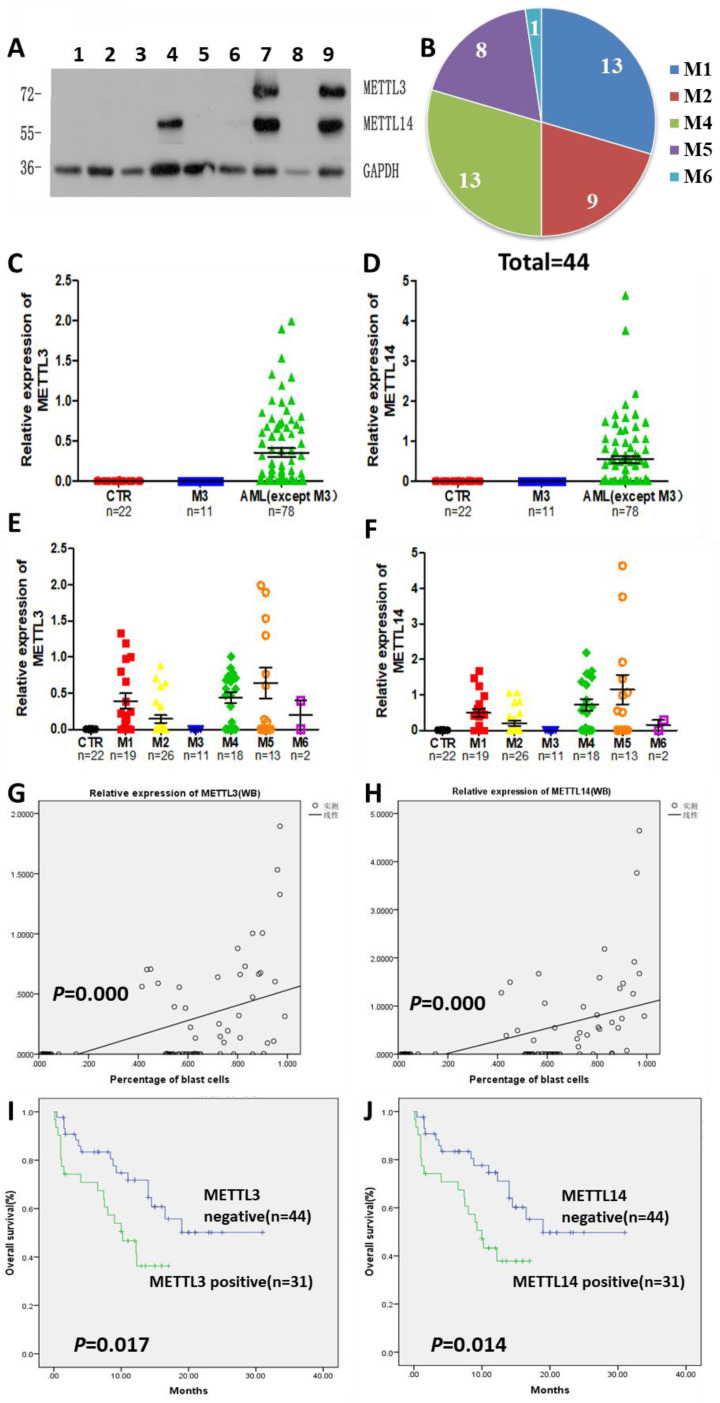
** High levels of METTL3 and METTL14 are associated with shorter survival in patients with AML.** A. Western Bolt assays showed the expression of METTL3, METTL14 and GAPDH (loading control) in some clinical samples (lane 1-3: control, lane 4-5: one AML-M4 patient in newly diagnosis [lane 4] and after [lane 5] chemotherapy, lane 6: AML-complete remission [CR)], lane 7: M2 relapse, lane 8: M3, lane 9: M1 at diagnosis); B. Case distribution of positive METTL3/14 patients according to the AML subtypes; C and D. The protein levels of METTL3 and METTL14 in the mononuclear cells of healthy subjects and patients with AML from the Western Blot assays were quantified using ImageJ. E and F. Relative protein levels of METTL3 and METTL14 in the mononuclear cells of control, M3 and other AML subtypes. G and H. The correlation between relative protein levels of METTL3 or METTL14 and percentage of blast cells. I. Comparison of OS between METTL3-negative and -positive patients; J. Comparison of OS between METTL14-negative and -positive patients.

The expression of METTL3 and METTL14 was examined by Western Blot (Fig. 1A), and quantified using the ImageJ software: samples with expression lower than 0.01 were considered to be negative. Compared with 22 normal samples, METTL3 and METTL14 were highly expressed in most of the patients with AML except the APL patients who showed low levels of METTL3 and METTL14. The percentage of positive METTL3 expression was different in each AML subtype, and the order of positive rates from high to low was M5, M4, M1 and M2, respectively from 15,18,19 and 26 samples, one of the two samples from AML-M6 patients was positive and another was negative (Fig. 1B), while all the APL patients (M3 subtype) did not express METTL3, similarly to the leukemia cell lines (Fig. 1C and 1E). The expression of METTL14 showed a similar trend (Fig. S1), and they were simultaneously positive or negative in the same sample (Fig. 1D and 1F). Interestingly, there was an obvious change of METTL3 and METTL14 levels in the same patient when newly diagnosed, after remission, and relapsed: patients which were positive for METTL3 and METTL14 expression when newly diagnosed, became negative after remission with chemotherapy, and positive again after recurrence (Fig.S2).

We collected data from 89 patients with AML, 12 patients of them were excluded from the study since they gave up treatment or their data were incomplete, and the clinical data of the remaining 75 patients were listed in Table 1. After comparing the clinical characteristics of METTL3- and METTL14-positive and -negative patients, it was found that there was no difference between the two groups, except for the proportion of blast cells and the first course remission status in the newly diagnosed patients: the patients with high proportion of blast cells had higher expression of METTL3 and METTL14 (*P* = 0.000; Fig. 1G and 1H), METTL3- and METTL14 -negative patients were easier to achieve complete remission. Follow-up of the patients showed that the median overall survival (OS) was 14.5 months (range 0.3 to 31.0 months). Importantly, we found that METTL3-positive patients had shorter OS (10.23 months) compared to METTL3-negative patients (OS, 20.475 months), and this difference was significant (P = 0.017; Fig. 1I). A similar trend was observed in METTL14-positive patients (OS, 10.0 months) compared to METTL14-negative patients (OS, 20.467 months) and, again, this difference was significant (P = 0.014; Fig. 1J).

Together, the expression of METTL3 and METTL14 was high in most of the patients with AML, and high expression of METTL3 and METTL14 correlated to short survival.

### METTL3 and METTL14 promote cell proliferation and affect cell cycle in K562 and kasumi-1 cell lines

**Figure 2:**
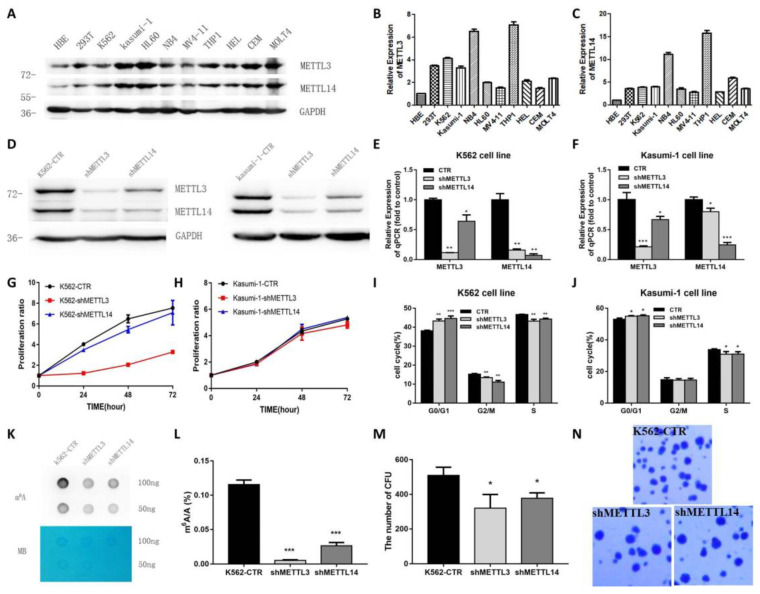
** METTL3 mainly promote cell proliferation and cell cycle in K562 and kasumi-1 cells.** A. Western Blot assays showed the levels of METTL3, METTL14 and GAPDH (loading control) in HBE, 293T, K562, kasumi-1, HL60, NB4, MV4-11, THP-1, HEL, CEM and MOLT4 cells. B and C. qPCR analysis of *METTL3* and *METTL14* expression in HBE, 293T, K562, Kasumi-1, HL60, NB4, MV4-11, THP-1, HEL, CEM and MOLT4 cells; D, E and F. The efficiency of *METTL3* and *METTL14* knockdown in K562 and kasumi-1 cells was confirmed by Western Blot (D) and qPCR (E and F); G. Cell proliferation was examined with an MTT assay; H and I. Cell cycle was measured by flow cytometry, and analyses were carried out with the ModiFit software. J. Dot blot assays of m^6^A mRNA in sh-control (shCTR), *METTL3-*silenced (shMETTL3), *METTL14-*silenced (shMETTL14) cells; methylene blue (MB) staining served as loading control; K. Analysis of m^6^A/A in mRNA of shCTR, shMETTL3, shMETTL14; L and M. The effects of *METTL3-* and *METTL14-*silence on anchorage-independent cell growth were examined in colony formation assays in K562 cells. Data are expressed as mean± SD, and experiments were performed in triplicate. *P<0.05; **P<0.01; ***P<0.001, with the Student's *t*-test. The results shown were representative of two independent experiments.

Next, the expression of METTL3 and METTL14 in leukemia cell lines was examined. We found that METTL3 and METTL14 were highly expressed in K562, kasumi-1, HL-60, NB4, MV4-11, THP-1, HEL, CEM and MOLT4 cell lines (Fig. 2A). The expression of METTL3 and METTL14 had a similar trend in these cell lines at both the mRNA and protein level (Fig. 2B and 2C).

To explore the role of METTL3 and METTL14, K562 and kasumi-1 cells were infected with lentiviral particles expressing shRNAs targeting METTL3 and METTL14. Upon RNA interference, the levels of METTL3 and METTL14 were reduced at both the protein and mRNA level (Fig. 2D, 2E and 2F). MTT assays showed that the knockdown of METTL3 and METTL14 inhibited cell proliferation, especially the knockdown of METTL3 (Fig.2G). Cell cycle analysis showed that the proportion of cells in G0/G1 phase increased, while cells in G2/M phase decreased after METTL3 and/or METTL14 knockdown (Fig.2H and 2I). The knockdown of METTL3, METTL14 reduced the levels of m^6^A in K562 cells compared with control, specifically the m^6^A/A ratio decreased by about 90%, 70%, respectively (*P*<0.001, Fig. 2J and 2K). Colony formation assays showed that the number of colonies decreased in K562 cells with METTL3 and/or METTL14 knockdown compared with the control group (Fig. 2L and 2M). These results indicated knockdown of METTL3 and METTL14 inhibits cell growth and colony formation.

### The knockdown of METTL3 and METTL14 promotes cell apoptosis and differentiation

The apoptosis of METTL3 and METTL14 knockdown cells slightly increased, though there was no any significant difference compared with the control groups (Fig.S3). Meanwhile, after treatment with 0.5μM DNR (daunorubicin) for 48 hours, apoptosis was significantly higher in METTL3- and METTL14-knockdown K562 cells, the percentage rates were 2.57%, 5.21% and 7.66% in control, METTL3- and METTL14-knockdown, respectively (P< 0.001; Fig. 3A left and 3D), while 0.60%, 1.62% and 3.53% after the treatment with 2.5μM 5-Azacitidine (5-Aza, Sigma) for 48 hours (*P*<0.001; Fig.3A right). Meanwhile, the apoptotic rates in METTL3 and METTL14- knockdown kasumi-1 cells were 30.9%, 31.65% and 33.95% after 0.5uM DNR treatment for 24 hours (*P*< 0.1; Fig. 3C left), while 16.05%, 22.15% and 20% after 500nM 5-Azacitidine treatment for 48 hours (*P*< 0.01; Fig. 3C right).

The effect of METTL3 and METTL14 on cell differentiation was analyzed by detecting the number of CD11b and CD14 positive cells. The results showed that the differentiation had no difference in METTL3- and METTL14-knockdown K562/Kasumi-1 cells and shRNA control cell, both untreated (Fig S3). Upon treatment with 1μM ATRA for 96 hours, the percentage of CD11b^+^ cells significantly increased by 2-3 folds (*P*<0.05), while the percentage of CD14^+^ cells was still similar to that in the control. However, after the treatment with 1μM PMA for 48 hours, the percentages of CD14^+^ cells were 3.33%, 14.05% and 13.70 %, respectively in control, METTL3- and METTL14-knockdown K562 cells, respectively (*P*<0.01. Fig. 3E and 3F). After treatment with 400nM DNR for 48 hours and 4uM 5-Azacitidine for 48 hours, the percentage of CD11b^+^ and CD14^+^cells also increased. In kasumi-1 cells knockdown with METTL3 and METTL14, the percentage of CD11b^+^ cells increased after treatment with 1μM ATRA for 96 hours and 5uM 5-Azacitidine for 24 hours, while the percentage of CD14^+^ cells decreased after treatment with 200ng PMA for 48 hours and 5uM 5-Azacitidine for 24 hours. The percentage of CD11b^+^ and CD14^+^cells had no significant difference after treatment with 500nM DNR for 24 hours (Fig. 3G and 3H). These results indicated that the knockdown of METTL3 and METTL14 promotes apoptosis and differentiation in leukemia cells.

### Identification of METTL3 and METTL14 potential targets

**Figure 4:**
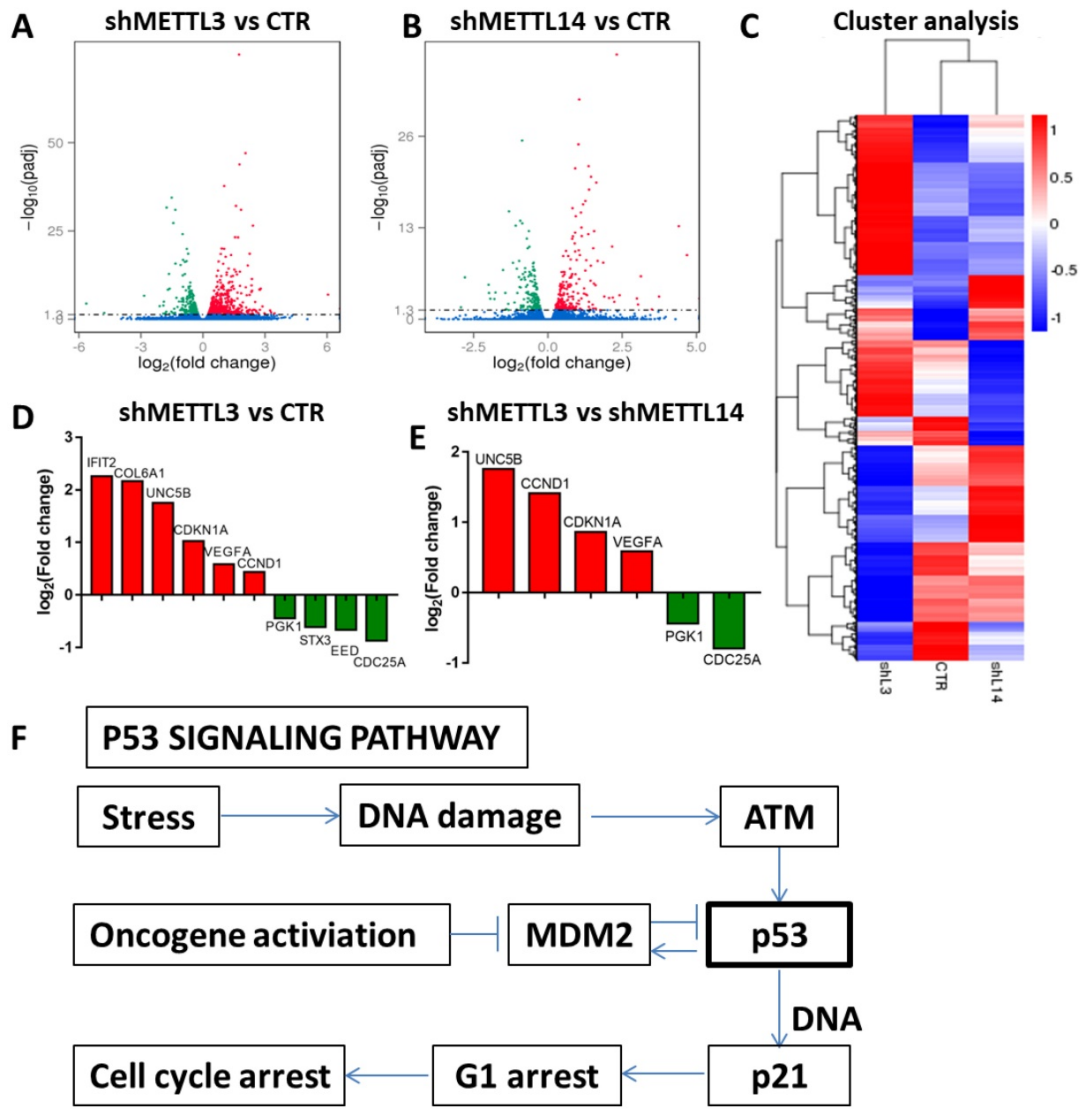
** RNA-seq to identify potential targets of METTL3 and METTL14.** A and B. Volcano map showing the fold changes of the genes differentially expressed upon *METTL3* or *METTL14* knockdown; red represents up-regulation, green represents down-regulation; C. Cluster analysis of differentially expressed genes; D and E. Histogram of the fold changes of the genes related to the p53 pathway upon *METTL3* or *METTL1 4* knockdown. F. The scheme of p53 signaling pathway.

The results above indicated that METTL3 and METTL14 play an important role in maintaining leukemic cells in an undifferentiated state. Next, we investigated the mechanism through which METTL3 and METTL14 play their function. RNA sequencing was used to identify potential targets of METTL3 and METTL14. The results showed that 578 genes were up-regulated and 480 genes were down-regulated after METTL3 knockdown, while 235 genes were up-regulated and 210 genes were down-regulated up after METTL14 knockdown in K562 cells compared with control (Fig. 4A and 4B). Among these, there were p53-related genes such as cyclin dependent kinase inhibitor 1A (*CDKN1A*, also called p21) and cyclin D1 (*CCND1*), which indicated that p53 signaling pathway was involved in the process (Fig. 4F). RNA-seq data also showed that the genetic changes between METTL3 and METTL14 knockdown in K562 cells are not exactly the same, for example, compared with knockdown of METTL14, CDKN1A and CCND1 were up-regulated after METTL3 knockdown in K562 cells (Fig. 4D and 4E), indicating that the function of METTL3 and METTL14 might be slightly different. GO analysis showed METTL3 and METTL14 had similar function in cytoplasm and protein binding (Fig. S4).

### The m^6^A of MDM2 mRNA play important roles in p53 pathway associated AML

**Figure 5:**
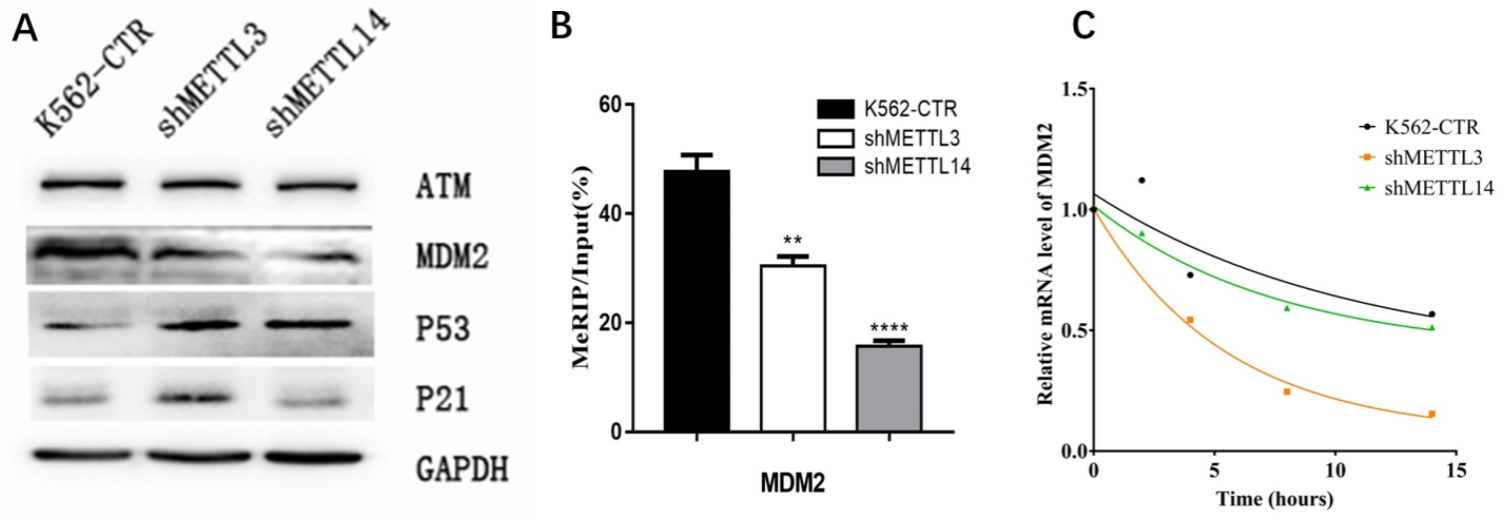
** The m6A of MDM2 mRNA play important roles in p53 pathway associated AML.** A. Expression of key proteins in p53 signal pathway examined by Western Blot in control (K562-CTR), METTL3-silenced (shMETTL3), *METTL14-*silenced (shMETTL14) in K562 cell lines; B. The m^6^A levels of mdm2 mRNA measured by M6A-RIP/MeRIP assay. C. the mRNA half-life (*t*_1/2_) of mdm2. The results shown were representative of three independent experiments.

To confirm the RNA-seq results, the expression of key proteins in the p53 pathway was detected by Western Blot. First, we checked p21 expression. Consistent with RNA-seq results, the expression of p21 was significantly elevated in METTL3-silenced K562 cells compared with control, while knockdown of METTL14 did not have this effect (Fig.5A). Surprisingly, we found the p53 expression increased after knockdown of METTL3 and METTL14. These results make us wonder whether there is any change of p53 upstream gene to prompt p53 expression? Therefore, we next analyzed the expression of upstream genes in the p53 pathway by Western Blot assay, including mouse double minute 2 (MDM2) and ATM. The results showed the expression of MDM2 not ATM was down-regulated after knockdown of METTL3 or METTL14 in K562 cell lines (Fig. 5A). This data indicated that MDM2, located in the upstream of the p53 pathway, may play an important role in METTL3- and METTL14-mediated development of leukemia.

As METTL3 and METTL14 were the component of m^6^A methyltransferase complex, we speculated mdm2 mRNA might be the target of m^6^A, high expression of METTL3 and METTL14 in AML cause the more m^6^A in mdm2 mRNA and more expression of MDM2 protein, at last, inhibit the p53 and downstream. Therefore, the m^6^A level of mdm2 mRNA was measured by the combination of m^6^A-RIP and mdm2 qPCR. The results showed the m^6^A level of mdm2 mRNA was significant lower after knock-down of METTL3 and METTL14 compared with control in K562 cell line (Fig. 5B), confirming our speculation. It was reported that mRNA stability may change and affect its expression after m^6^A methylation. Therefore, we detected the half-life of mdm2 mRNA under actinomycin-D treatment and the results showed that the half-life of mdm2 became shorter than control cells after knock-down of METTL3 and METTL14 in K562 cell line (Fig. 5C). All these results suggested that the low stability of mdm2 mRNA transcripts upon the lower m^6^A levels mediated by METTL3- and METTL14-knockdown could lead to the activation of p53 signal pathway, in the end, reverse partially the process of leukemia.

## Discussion

Our study showed that METTL3 and METTL14 are highly expressed in patients with AML (except APL), and high levels of METTL3 and METTL14 correlate to shorter survival, indicating that METTL3 and METTL14 are adverse factors in patients with AML. In leukemia cell lines, knockdown of METTL3 or METTL14 inhibited cell proliferation, induced cell cycle arrest and decreased the ability of the cells to form colonies. Moreover, METTL3- and/or METTL14- knockdown was associated with increased apoptosis after the treatment with DNR or 5-Aza, and differentiation into granulocytes upon ATRA treatment, and mononuclear cells upon PMA treatment. These results indicate that m^6^A RNA methylation plays an oncogenic role in leukemogenesis.

It has been reported that silencing of METTL3 in human hematopoietic stem/progenitor cells and human myeloid leukemia cells promotes cell differentiation and apoptosis, while overexpression of METTL3 has opposite effects 20. Another study has found that METTL14 is highly expressed in AMLs and down-regulated during normal myelopoiesis: the depletion of METTL14 would promote the differentiation of human hematopoietic stem/progenitor cells and AML cells into myeloid cells 24. These observations are consistent with the results of our study and suggest that both METTL3 and METTL14 play an oncogenic role in AML.

Clinically, most AML cases have a p53 dysfunction not associated with gene mutation that leads to DNA damage accumulation; in such way, hematopoietic stem cells might become leukemia stem cells 32. An analysis of the Cancer Genome Atlas research network (TCGA) database in AML has shown that mutations and/or copy number variations of m^6^A regulatory genes are associated with *TP53* mutations in patients with AML; among these, the loss of ALKBH5 copy number is the most common, and has a striking relation with *TP53*^33^-^34^. In addition, a study in HepG2 cells has revealed that the deletion of METTL3 results in the change of more than 20 p53-related genes such as *MDM2*, mouse double minute 4 (*MDM4*) and p21, resulting in instability and reduced function off p53 35. Zhao et al explored the regulatory role of m^6^A on p53 activation using an arsenite-transformed keratinocyte model, the HaCaT-T cell line. They found that the cells exhibited an increased m^6^A level along with an aberrant expression of the methyltransferases, demethylase, and readers of m^6^A. Knockdown of the m^6^A methyltransferase METTL3 significantly decreased m^6^A level, restoring p53 activation and inhibiting cellular transformation phenotypes in the arsenite transformed cells. Further, using both a bioinformatics analysis and experimental approaches, they demonstrated that m^6^A downregulated the expression of the positive p53 regulator PRDM2 through the YTHDF2-promoted decay of PRDM2 mRNAs, and upregulated the expression of the negative p53 regulator YY1 and MDM2 through YTHDF1-stimulated translation of YY1 and MDM2 mRNA 36. Ghazi et al found Fusaric acid, a food-borne mycotoxin, epigenetically decreased p53 expression in HepG2 cells by reducing METTL3 and METTL14 expression, and ultimately reduced p53 translation 37. Our results revealed that the knockdown of METTL3 and METTL14 leads to multiple changes in the p53 pathway, including the up-regulation of p53 and *CDKN1A* (*p21*), and the lengthened half-life of mdm2 mRNAs. These results suggest that m^6^A methylation may affect the p53 pathway in leukemic cells and mediate the development of leukemia. To date, similar results have not been reported in AML.

It has been reported that p53 mutation or abnormal function is closely related to the maintenance of stem cells. Lin *et al.* have found that, in human embryonic stem cells, low activity of p53 is associated with an undifferentiated state, and activation of p53 by phosphorylation inhibits the expression of the core transcription factor Nanog and promotes differentiation, while re-inhibition of p53 function causes the reprogramming of the differentiated cells to the embryonic state 38. Meanwhile, the down-regulation of p53 and its downstream effector p21 promotes the formation of inducible pluripotent stem cells 39,40. These results suggest that the p53 pathway is important for embryonic stem cell differentiation, and its inactivation can maintain the embryonic stem cells in an undifferentiated state. On the other hand, recent studies have shown that tumor stem cells may be obtained by reprogramming differentiated cells. This process relies on the same signaling pathways activated in the generation of inducible pluripotent stem cells 41. Kitajima* et al.* have found that knockout of the p53, retinoblastoma (*Rb*) and *N-ras* genes induces the formation of cancer-like stem cells, which express embryonic stem cells genes, have tumor cell properties, and are multipotent 42. Notably, the reactivation of the p53-Rb-Nras pathway induces the differentiation of these cancer-like stem cells and inhibits their ability to invade and metastasize 43. These data suggest that inactivation of the p53 pathway plays an important role in tumor stem cell formation and tumor stability.

Therefore, we assumed that the high expression of METTL3 and METTL14 in AML increases the levels of m^6^A in mdm2 mRNAs, an upstream inhibitor of p53, causes its expression, lead to inactivation of the p53 pathway, at last resulting in the maintenance of a population of leukemic stem cells.

In summary, our study demonstrates that both METTL3 and METTL14 play an oncogenic role in AML by increasing the m^6^A levels in mdm2 mRNA and in targeting mdm2/p53 signal pathway.

## Supplementary Material

Supplementary figures.

## Figures and Tables

**Figre 3:**
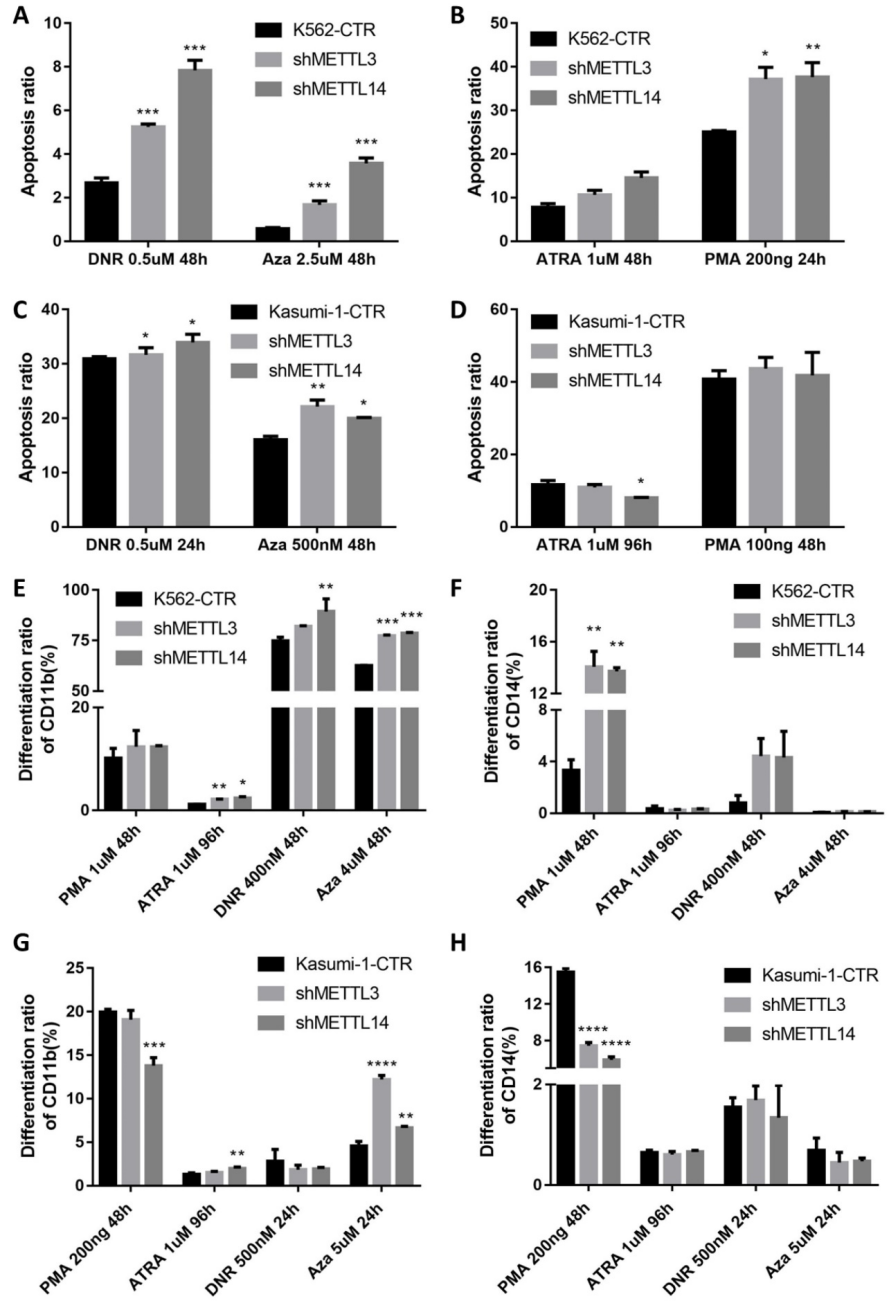
** The knockdown of *METTL3* and *METTL14* induces apoptosis and differentiation.** A and B. Apoptosis in K562-CTR, shMETTL3, shMETTL14 after the treatment with DNR/Aza (A) or ATRA/PMA (B). C and D. Apoptosis in kasumi-1-CTR, shMETTL3, shMETTL14 after the treatment with DNR/Aza (C) or ATRA/PMA (D) and Aza. E and F. Percentage of the CD11b^+^(E) and/or CD14^+^(F) population after the treatment with ATRA, PMA, DNA and Aza. G and H. Percentage of the CD11b^+^(G) and/or CD14^+^(H) population after the treatment with ATRA, PMA, DNA and Aza. Apoptosis and expression of CD11b and CD14 were examined by flow cytometric, and the data are expressed as the mean± SD. Experiments were performed in triplicate. *P<0.05; **P<0.01; ***P<0.001, with the Student's *t*-test.

**Table 1:** Characteristics of 75 AML patients in METTL3/14 negative and positive group

Patient characteristics	Values (negative)	Values (positive)	*P* value
Gender (M/F)	23/21	18/13	0.620
Age at diagnosis (≥60/<60)	7/37	7/24	0.465
Risk stratification (favorable/intermediate/poor)	22/8/14	8/8/15	0.108
WBC counts (≥100/<100)	6/38	8/23	0.183
Blasts (≥50%/<50%)	26/18	27/4	**0.010**
LDH (≥300/<300)	24/20	19/12	0.561
3 months status (alive/death)	38/6	23/8	0.183
6 months status (alive/death)	33/11	23/8	0.937
12 months status (alive/death)	22/22	12/19	0.333
first course remission (CR/NR)	30/14	13/18	**0.024**
FLT3 mutation (+/-)	10/34	9/22	0.536
NPM1 mutation (+/-)	8/36	4/27	0.539
DNMT3A mutation (+/-)	7/37	7/24	0.465
TET2 mutation (+/-)	16/28	14/17	0.444
IDH mutation (+/-)	4/40	2/29	1.000
CEBPA mutation (+/-)	10/34	6/25	0.726
ASXL1 mutation (+/-)	14/30	12/19	0.537

M: male; F: female; CR: complete remission; NR: no response.

## Abbreviations

m^6^A: N6-methyladenosine
AML: acute myeloid leukemia
METTL3/14: methyltransferase-like three/fourteen
WTAP: Wilms' tumor 1-associated protein
FTO: fat mass and obesity-associated protein
ALKBH5: AlkB homologue 5
YTHDF: YT521-B homology domain family
ASB2: ankyrin repeat and SOCS box protein 2
RARA: retinoic acid receptor alpha
CDKN1A/p21: cyclin dependent kinase inhibitor 1A
OS: overall survival
GAPDH: glyceraldehyde 3-phosphate dehydrogenase
CR: complete remission
DNR: daunorubicin
MDM2: mouse double minute 2
