# Supplementary material for: The m6A RNA methyltransferase METTL3/METTL14 promotes leukemogenesis through the mdm2/p53 pathway in acute myeloid leukemia
Source: J Cancer. 2022 Jan 4;13(3):1019–30. doi: 10.7150/jca.60381 (PMC8824895; doi:10.7150/jca.60381)
Supplement: Supplementary file 1 — Supplementary figures. [file jcav13p1019s1.pdf]

Supplementary figures

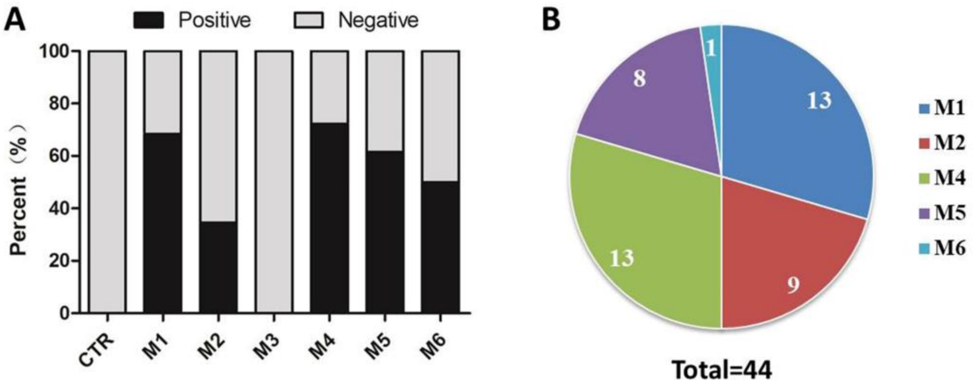

Fig. S1 The distribution of METTL3 and METTL14 in different FAB AML subtypes. A. METTL3/14 expression in mononuclear cell of normal and AML patients. **B.** Case distribution of positive METTL3/14 patients according to the AML subtypes.

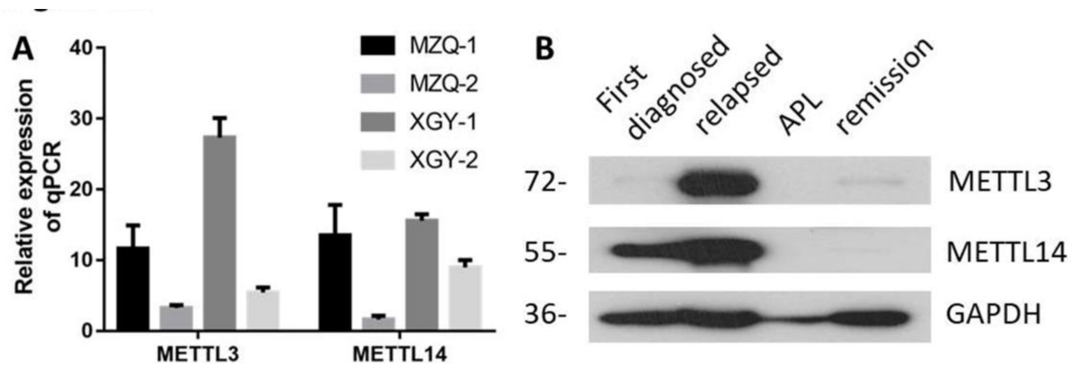

Fig. S2 The differences in METTL3 and METTL14 during remission and relapsed AML. A. qPCR analysis of *METTL3* and *METTL14* expression in MZQ-1, MZQ-2, XGY-1, XGY-2 (MZQ-1, XGY-1: Pre-treatment; MZQ-2, XGY-2: Post-treatment); B. Western Bolt assays showed the expression of METTL3, METTL14 and GAPDH (loading control) in some clinical samples (lane 1: first diagnosed AML, lane 2: relapsed AML, lane 3: APL; lane 4: remission AML).

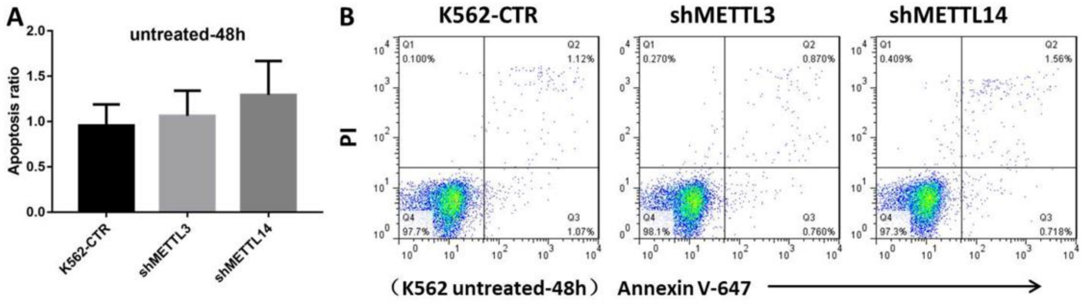

Fig. S3 The apoptosis of *METTL3* and/or *METTL14* knockdown cells slightly increased. A and B. Apoptosis were examined by flow cytometry in untreated control (K562-CTR) K562 cells, *METTL3* knockdown (shMETTL3) and *METTL14* knockdown (shMETTL14) cells for 48h， and the data are expressed as the mean ± SD. Experiments were performed in triplicate. *P<0.05; **P<0.01; ***P<0.001, with the Student's *t*-test

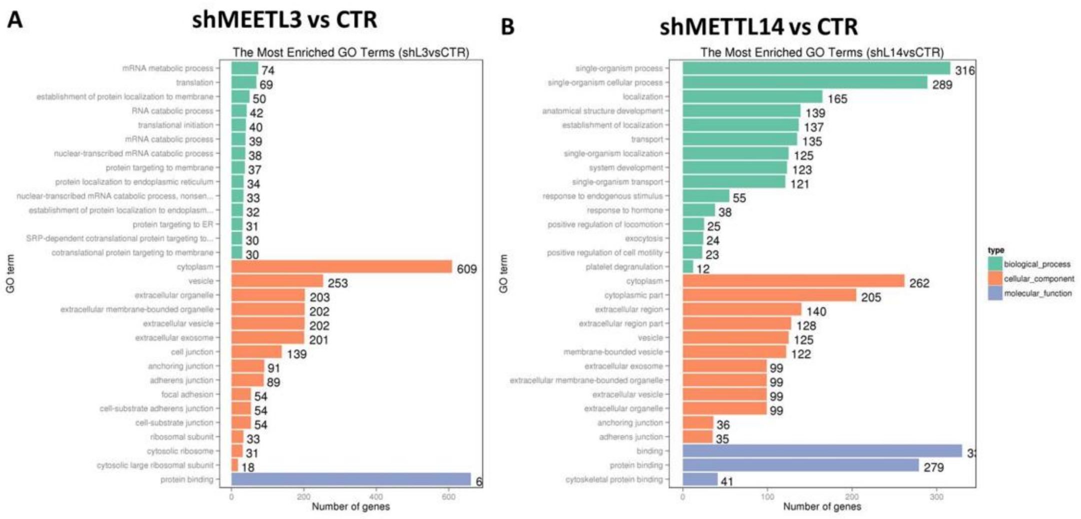

Fig. S4 GO analysis in METTL3/METTL14 knockdown cells. A and B. The GO analysis upon *METTL3*(A) or *METTL14*(B) knockdown; green represents biological process, red represents cellular component, blue represents molecular function.
